# Synthesis and Near Infrared Luminescence Properties of a Series of Lanthanide Complexes with POSS Modified Ligands

**DOI:** 10.3390/molecules24071253

**Published:** 2019-03-30

**Authors:** Qingrui Zhang, Xiuyun Yang, Ruiping Deng, Liang Zhou, Yang Yu, Yunhui Li

**Affiliations:** 1 School of Chemistry and Environmental Engineering, Changchun University of Science and Technology, Changchun 130000, China; qrzhang784783@126.com (Q.Z.); liyh@cust.edu.cn (Y.L.); 2 Changchun Institute of Applied Chemistry, Chinese Academy of Sciences, Changchun 130022, China; yuy@ciac.ac.cn

**Keywords:** lanthanide complexes, polyhedral oligomeric silsesquioxanes, near-infrared luminescence

## Abstract

A polyhedral oligomeric silsesquioxanes (POSS) modified 8-hydroxyquinoline derivative (denoted as Q-POSS) was synthesized and used as a ligand to coordinate with lanthanide ions to obtain a series of lanthanide complexes Ln(Q-POSS)_3_ (Ln = Er^3+^, Yb^3+^, Nd^3+^). The as-prepared lanthanide complexes have been characterized by FT-IR, UV–Vis, and elemental analysis. All these complexes showed the characteristic near-infrared (NIR) luminescence originated from the corresponding lanthanide ions under excitation. Compared with the unmodified counterparts LnQ_3_ (HQ = 8-hydroxyquinoline), the Ln(Q-POSS)_3_ complexes showed obviously increased emission intensity, which was ascribed mainly to the steric-hindrance effects of the POSS moiety in the ligands. It is believed that the POSS group could suppress undesired excimer formation and intermolecular aggregation, thus decreasing the concentration quenching effect of the corresponding lanthanide complexes.

## 1. Introduction

Luminescent lanthanide (Ln) ions have attracted scientific and technological interest due to their unique photophysical properties and potential applications in the fields of cell imaging [1], optical amplification [2], light-emitting diodes [3], luminescent probes [4], etc. However, there are still some challenges for the application of the Ln complexes. Resulting from the weak absorption efficiency of f–f transitions in Ln^3+^ ions [5], designing efficient sensitization systems for lanthanide-based NIR emitters remains a challenge. Moreover, many Ln complexes suffer from the poor thermal stability. To resolve the as-mentioned problems, many efforts have been devoted to the design and synthesis of lanthanide complexes with efficient energy transfer ligands. Quinoline is one of the most popular ligands for lanthanide ions, thanks to the excellent complexation properties of the quinoline molecule, which has been selected for NIR luminescent studies as well [6,7,8]. In addition, the introduction of Ln complexes into the inorganic matrix, either the fabrication of the organic/inorganic hybrid materials by chemical bonds or by physical interaction, has been proved to be a highly effective method to improve the thermal stability of Ln complexes [9,10].

Among the hybrid material families, polyhedral oligomeric silsesquioxanes (POSS), a class of inorganic/organic hybrids at the molecular level, has gained many interests in the materials field due to its well-defined 3D structures, good biocompatibility [11], and good solubility in organic solvents [12]. With a generic empirical formula (RSiO_1.5_)_n_ (where n is commonly 6, 7, 8, 10, or 12), it is comprised of an inner inorganic core and external substituent groups, and the sizes of the building blocks range from 1 to 3 nm, which can be considered as a class of sphere-like nanomaterials [13]. When POSS units were introduced into organic or polymeric materials, glass transition temperature, mechanical strength, and thermal stability of the corresponding materials were enhanced as well [14,15]. POSS have shown a great potential in the Ln-complexes-based hybrid materials recently, and Li and Sun et al. had reported excellent luminescent POSS modified Ln complexes [16,17].

Here, we designed a novel ligand using POSS to modify the 8-hydroxyquinoline core to prepare the hybrid lanthanide complexes. Detailed NIR luminescent properties of these hybrid materials were investigated. It has been found that the Ln(Q-POSS)_3_ complexes show obviously enhanced emission intensity when they are compared with their counterparts LnQ_3_, which is discussed in the steric hindrance effects of the POSS and the change of the energy level of the modified ligand.

## 2. Results

**Scheme 1:**
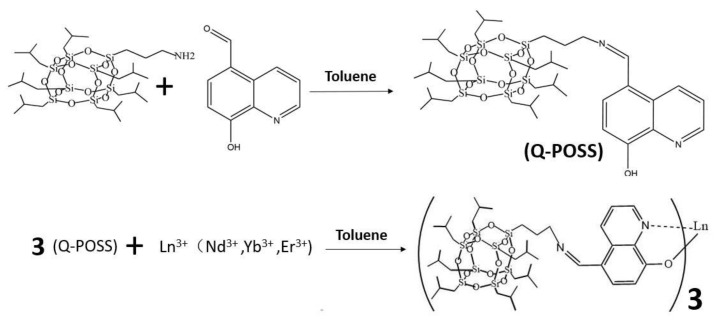
The synthetical procedure of ligands POSS modified 8-hydroxyquinoline derivative (Q-POSS) and Ln complexes Ln(Q-POSS)_3_.

The synthetical procedure of the ligand Q-POSS and the Ln complexes Ln(Q-POSS)_3_ (Ln = Er^3+^, Nd^3+^, Yb^3+^) is depicted in Scheme 1. The structure characterization of the ligand and complexes were carried out by ^1^HNMR, MS, elemental analysis and FT-IR methods. The ^1^HNMR and MS spectra (Figures S1 and S2) proved that the ligand Q-POSS was synthesized successfully.

### 2.1. FT-IR Analysis

**Figure 1:**
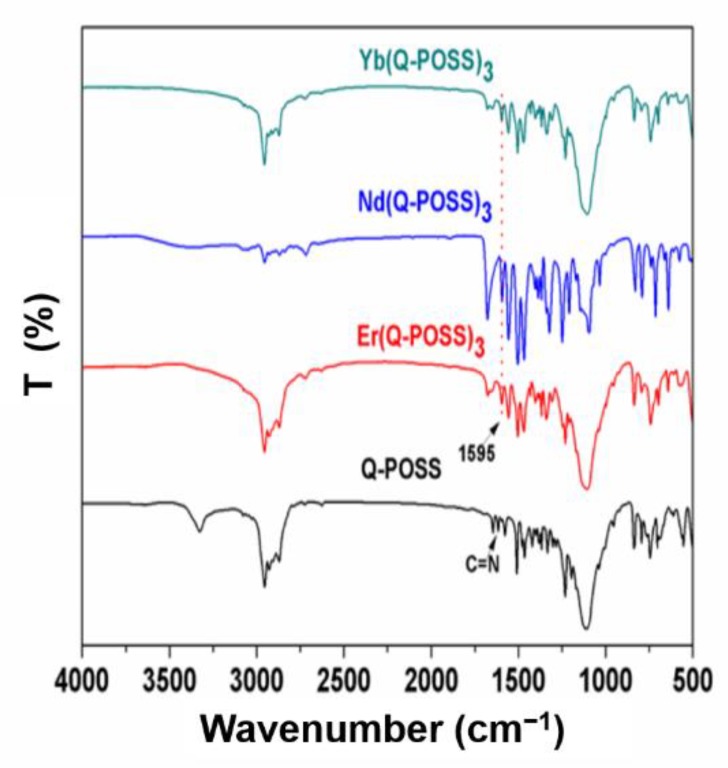
The FT-IR spectra of Yb(Q-POSS)_3_, Nd(Q-POSS)_3_, Er(Q-POSS)_3_, and Q-POSS.

The FT-IR spectra of Yb(Q-POSS)_3_, Nd(Q-POSS)_3_, Er(Q-POSS)_3_, and Q-POSS are shown in Figure 1. The absorption peak at around 1100 cm^−1^ for Yb(Q-POSS)_3_, Nd(Q-POSS)_3_, Er(Q-POSS)_3_, and Q-POSS, respectively, represents the vibrations of the siloxane Si-O-Si groups and is a general feature of POSS derivatives [18]. The characteristic bands at 2870–2954 cm^−1^ were clearly observed, which represent the vibrations of −CH_3_ and −CH_2_. In Yb(Q-POSS)_3_, Nd(Q-POSS)_3_, and Er(Q-POSS)_3_, the peak for −OH group at 3326 cm^−1^ disappeared completely, and the stretching vibration peak of C=N bond shifted from 1615 to 1595 cm^−1^, indicating that the nitrogen and oxygen atom in quinoline moiety participates in coordination with Ln ions [19].

### 2.2. UV–Vis Analysis

**Figure 2:**
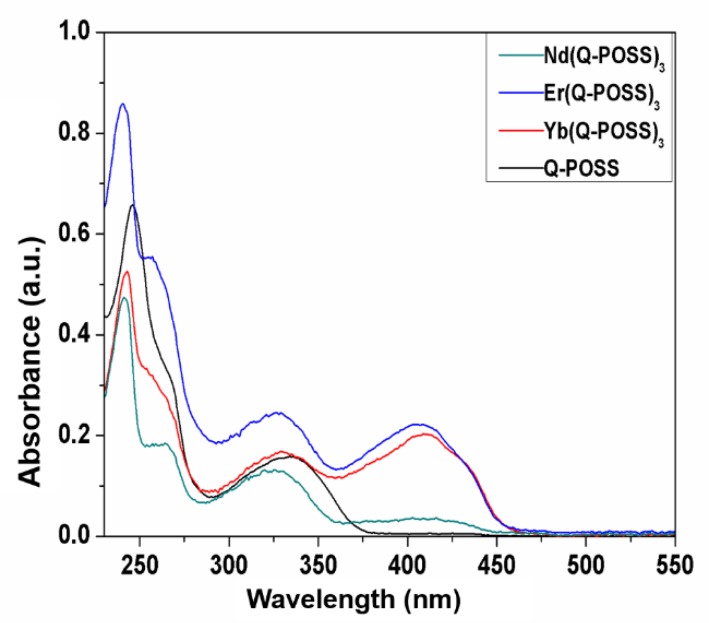
UV–Vis absorption spectra of Q-POSS, Er(Q-POSS)_3_, Nd(Q-POSS)_3_, and Yb(Q-POSS)_3_ in dichloromethane solutions (1 × 10^−5^ M) at room temperature.

Figure 2 displays the UV−Visible (UV−Vis) absorption spectra of the solutions of Q-POSS, Er(Q-POSS)_3_, Nd(Q-POSS)_3_, and Yb(Q-POSS)_3_ dissolved in dichloromethane (1 × 10^−5^ M), respectively. The absorption peaks for Q-POSS appeared at 246 and 334 nm, respectively, which can be attributed to the π–π* transitions of quinoline moiety. For Er(Q-POSS)_3_, Nd(Q-POSS)_3_, and Yb(Q-POSS)_3_ complexes, they show a strong absorption band at about 241 nm and two weak absorption bands at around 330 and 409 nm, in which the absorption bands at 241 nm and 330 nm can be ascribed to the spin-allowed π–π* transitions of the ligands, and the band at 409 nm can be assigned to both spin–orbit coupling enhanced (π–π*) and spin-forbidden LMCT transitions [20]. The UV–Vis absorption spectra of Q, ErQ_3_, NdQ_3_, and YbQ_3_ in dichloromethane solutions (1 × 10^−5^ M) at room temperature are shown in Figure S3. They show similar absorption behaviors with that of the Q-POSS and Ln(Q-POSS)_3_. The absorption peaks for the unmodified ligand 8-hydroxyquinoline (HQ) appeared at 241 and 317 nm, respectively. Besides the π–π* transitions of the ligands, occurring at 242 and 317 nm, respectively, the LnQ_3_ complexes show weak bands at about 385 nm, which are also the spin–orbit coupling enhanced (π–π*) and spin-forbidden LMCT transitions, as mentioned in the cases of Ln(Q-POSS)_3_.

### 2.3. Luminescent Properties

**Figure 3:**
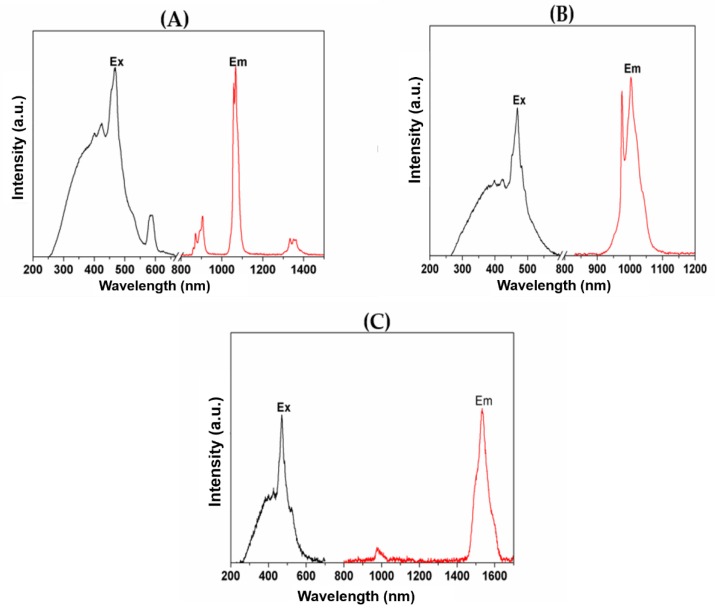
Excitation and emission spectra of Ln(Q-POSS)_3_. (**A**) Nd (Q-POSS)_3_ (λ_ex_ = 469 nm, λ_monitor_ = 1068 nm), (**B**) Yb (Q-POSS)_3_ (λ_ex_ = 469 nm, λ_monitor_ = 980 nm), and (**C**) Er(Q-POSS)_3_ (λ_ex_ = 471 nm, λ_monitor_ = 1536 nm).

The Luminescent properties of the Ln(Q-POSS)_3_ (Ln = Nd^3+^, Yb^3+^, Er^3+^) complexes in the solid state were investigated. Figure 3 displays their excitation and emission spectra. All these complexes exhibit similar excitation bands, with a broad band ranging from 250 to 560 nm, which arise from the 8-hydroxyquinolinate ligand related transitions and the LMCT from the complexes according to the UV–Vis absorption spectra as mentioned above. And the main excitation peak occurs at about 469 nm. For the case of Nd(Q-POSS)_3_, the excitation band is superimposed with the characteristic absorption transition ^4^I_9/2_→^2^G_7/2_ (585 nm) of the Nd^3+^ ion as well. After excitation, all the Ln(Q-POSS)_3_ (Ln = Nd^3+^, Yb^3+^, Er^3+^) complexes show the characteristic transitions of the corresponding Ln^3+^ ion. The emission spectrum of Nd(Q-POSS)_3_ consists of three narrow bands from the f–f transitions of the Nd^3+^ ion in the range of 800–1700 nm, with the main band occurring at 1068 nm (^4^F_3/2_→^4^I_11/2_), and two other bands at 904 nm (^4^F_3/2_→^4^I_9/2_) and 1333 nm (^4^F_3/2_→^4^I_13/2_), respectively. The Yb(Q-POSS)_3_ complex emits in the range of 910–1100 nm, with a sharp band at around 980 nm assigned to the ^2^F_5/2_→^2^F_7/2_ transition of the Yb^3+^ ion and a broader vibronic component at a longer wavelength. The emission spectrum of Er(Q-POSS)_3_ is comprised of two bands from the Er^3+^ ion, which are assigned to the ^4^I_11/2_→^4^I_15/2_ (980 nm) and ^4^I_13/2_→^4^I_15/2_ (1536 nm) transitions, respectively.

**Figure 4:**
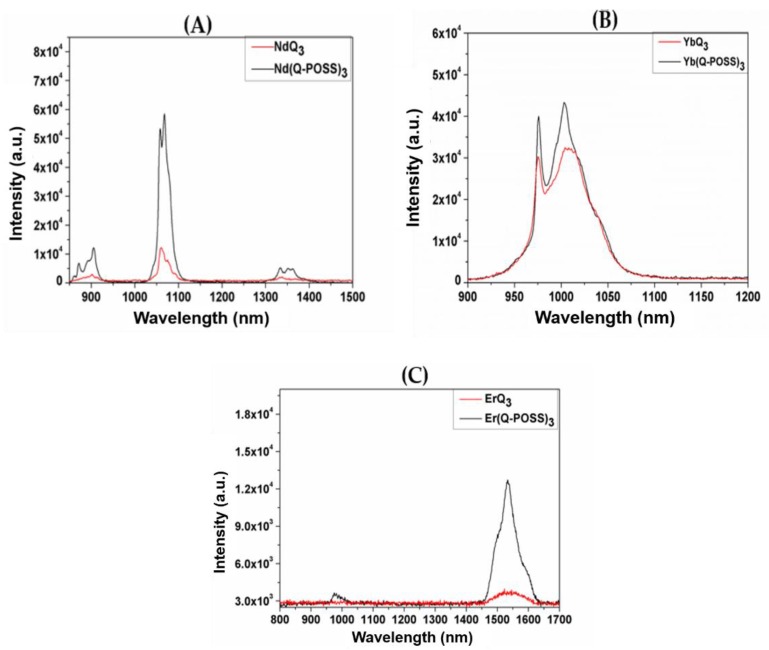
Emission spectra of the Ln(Q-POSS)_3_/LnQ_3_ couples. (**A**) Nd(Q-POSS)_3_ and NdQ_3_ (λ_ex_ = 469 nm), (**B**) Yb(Q-POSS)_3_ and YbQ_3_ (λ_ex_ = 469 nm), and (**C**) Er(Q-POSS)_3_ and ErQ_3_ (λ_ex_ = 471 nm).

To further investigate the luminescent properties of the Ln(Q-POSS)_3_ complexes, the complexes LnQ_3_ without POSS modification were also prepared to be used as the counterparts of the Ln(Q-POSS)_3_ complexes. We have measured the excitation spectra (Figure S4, S5, and S6 in Supporting Information) and emission spectra of the Ln(Q-POSS)_3_/LnQ_3_ couples in the solid state under the same condition. The comparison results of the emission spectra of Ln(Q-POSS)_3_/LnQ_3_ (Ln = Nd^3+^, Yb^3+^, Er^3+^) are displayed in Figure 4. In comparison with the complexes LnQ_3_ with unmodified ligand, the corresponding Ln(Q-POSS)_3_ (Ln = Er^3+^, Yb^3+^, Nd^3+^) complexes show obviously enhanced luminescence intensity.

## 3. Discussion

Ln complexes with emissions in the NIR region such as Nd^3+^, Yb^3+^, and Er^3+^ have been intensely studied in the past decades on account of their attractive features, such as large stock shift, emission line spectrum, ideal excitation and emission wavelengths, long fluorescence lifetime, and small external influence [21,22,23]. They showed special importance in the field of optical amplifier, laser, night vision, etc. For example, the 1.5 μm emission of Er^3+^ ion has been widely used in the IR laser operating at eye-safe wavelengths and in the fabrication of optical amplifiers [24]. The 1333 nm emission band of the Nd^3+^ ion also occurs at the second window of the optical amplifier. Therefore, it is significant for improving NIR luminescence of Ln complexes [25].

In this study, POSS modified ligand Q-POSS and unmodified ligand HQ were used to synthesize a series of Ln complexes. Nd(Q-POSS)_3_/NdQ_3_, Yb(Q-POSS)_3_/YbQ_3_, and Er(Q-POSS)_3_/ErQ_3_ exhibit similar band location in their excitation spectra (Figure S4, S5, and S6 in Supporting Information, respectively) and emission spectra (Figure 4), which are related to the 8-hydroxyquinolinate based excitation and the f–f transitions from the center Ln^3+^ ions, respectively. But tiny differences between the emission spectra of the Ln(Q-POSS)_3_/LnQ_3_ could still be observed; for example, the shapes of the peaks of the Nd^3+^ ion at 1068 nm (^4^F_3/2_→^4^I_11/2_), which may be because that the tiny difference of the coordination environments differs from the Ln^3+^ ion when it is coordinated with the ligands of Q-POSS and Q. What should be noted is that the excitation and emission intensity of the Ln(Q-POSS)_3_ are higher than that of the LnQ_3_, which reveals that the introduction of the POSS moiety into the ligand could provide better luminescent properties for the resulted complexes. It is believed that the enhancement of the luminescence intensity is related to the steric-hindrance effects of the POSS moiety in the ligands. The large volume of the POSS group in the Ln(Q-POSS)_3_ will decrease the concentration quenching effect of the corresponding Ln complexes, and suppress undesired excimer formation and intermolecular aggregation [26].

**Scheme 2:**
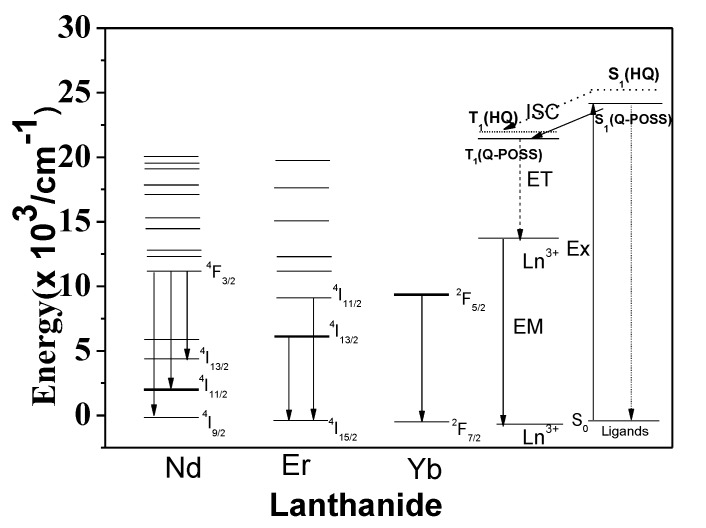
Schematic energy level diagram of the emissive states of the Ln ions (Ln = Er^3+^, Nd^3+^, and Yb^3+^) compared with the triplet states of the Q-POSS and 8-hydroxyquinoline (HQ).

Another possible factor for the emission intensity enhancement is the change of energy levels of the ligands, which will affect the energy transfer efficiency from the ligand to the center Ln^3+^ ion, and, accordingly, the overall photoluminescence performance. In order to investigate the energy transfer from the Q-POSS ligands to the lanthanide ions, Gd complex Gd(Q-POSS)_3_ was prepared and used to study the triplet energy level of the ligand since Gd has no energy levels below 312 nm (32000 cm^−1^) and it cannot accept energy from the ligands. The phosphorescence spectrum of the Gd(Q-POSS)_3_ complex (Figure S7 in Supporting Information) at 77 K under UV excitation was obtained. The first phosphorescence peak occurred at 469 nm; thus, the triplet energy level of the Q-POSS was calculated to be 21322 cm^−1^. According to the reported literature, the triplet energy level of GdQ_3_ was located at 21830 cm^−1^ [19]. In other words, the triplet energy level of our modified ligand Q-POSS is a bit lower than that of the ligand HQ. As there is no conjugation effect between the POSS cage and the core ligand 8-hydroxyquinoline, the −C=N group at 5-position of 8-hydroxyquinoline moiety is ascribed to the reduction of the energy level of the Q-POSS. As shown in Scheme 2, compared with that of the ligand HQ, the energy level of the Q-POSS is closer to the 4f excited state energy level of the lanthanide ions (Er^3+^, Nd^3+^, Er^3+^), indicating a possible more efficient energy transfer between the ligand and the lanthanide ions, thus a better performance in the characteristic emission of lanthanide ions. However, the difference between the energy levels of the Q-POSS and HQ is not that large, which could also be verified from the small differences of the absorption peaks originated from the ligands of the Q-POSS and HQ (246 and 334 nm for Q-POSS and 241 and 317 nm for HQ, respectively); thus, such small change of electronic energy level can’t be the main cause of the enhanced emission intensity of the Ln(Q-POSS)_3_. We believe that the luminescence optimization in the Ln(Q-POSS)_3_ comes mainly from the POSS moiety itself.

In summary, a novel ligand based on 8-hydroxyquinoline derivative modified by POSS has been synthesized, and a series of molecular hybrid NIR luminescent complexes Ln(Q-POSS)_3_ (Ln = Er^3+^, Yb^3+^, Nd^3+^) have been prepared. The optical properties of the as-prepared complexes have been investigated. After ligand-mediated excitation, all of the Ln(Q-POSS)_3_ (Ln = Er^3+^, Yb^3+^, Nd^3+^) complexes exhibited the characteristic NIR luminescence of the corresponding lanthanide ions. Compared with the complexes LnQ_3_ with unmodified ligand, the corresponding Ln(Q-POSS)_3_ (Ln = Er^3+^, Yb^3+^, Nd^3+^) have showed enhanced luminescence performances, which is mainly ascribed to the steric-hindrance effects of the POSS on the complexes. It is believed that the POSS modification will benefit for the preparation of the potential excellent lanthanide-based luminescent materials.

## 4. Materials and Methods

### 4.1. Drugs and Reagents

Aminopropylisobutyl POSS (Hybrid Plastics Co., Hattiesburg, MS, USA); 5-formyl-8-hydroxyquinoline (Shanghai SHUYA Co., Shanghai, China); toluene; methanol; erbium(III) chloride hexahydrate; ytterbium(III) chloride and neodymium(III) chloride (Aladdin Co., China); 8-hydroxyquinoline (Tianjin Chemical Reagent Research Institute, Tianjin, China); All the chemicals were analytical grade reagent.

### 4.2. Instruments

Excitation and emission spectra were measured with an Edinburgh FLSP 920 fluorescence spectrophotometer. FT-IR spectra were measured within a 4000–400 cm^−1^ region on an American BIO-RAD Company model FTS135 infrared spectrophotometer (Philadelphia, PA, USA) with the KBr pellet technique. ^1^HNMR spectra were obtained on the Avance III 400MHz. UV–Vis absorption was recorded on a Shimadzu UV-2550 spectrometer (Kyoto, Japan). The low temperature phosphorescence spectra of the Gd(Q-POSS)_3_ complexes were measured on a Hitachi F-4500 spectrophotometer at liquid nitrogen temperature (77 K). Elemental analysis was carried out on a Vario MICRO cube Elemental Analyzer. MALDI-TOF mass spectrum was obtained from Bruker microTOF Electro-spray Mass Spectrometry.

### 4.3. Synthesis of Aminopropylisobutyl POSS Modified 8-Hydroxyquinoline Hybrid Ligand (Q-POSS)

The 5-formyl-8-hydroxyquinoline and aminopropylisobutyl POSS were dissolved in a suitable round bottom flask of toluene with a molar ratio of 1:2, and then refluxed for 12 h under stirring in a nitrogen atmosphere at 90 °C. After that, the solvent was distilled off under reduced pressure, yielding the aminopropylisobutyl POSS modified 8-hydroxyquinoline: yellow solid (Q-POSS) in 53.1% yield (0.95 g). ^1^HNMR (CDCl_3_, δ, ppm): 9.78 (1H, d, *J* = 8.5 Hz), 8.83 (1H, d, *J* = 4.1 Hz), 8.60(1H, d), 7.70 (1H, d, *J* = 7.9 Hz), 7.56 (1H, dd), 7.19 (1H, d, *J* = 8.0 Hz), 3.65 (2H, t, −N-CH_2−_), 1.87 (7H, m, −CH−), 0.98 (42H, m, −CH_3_), 0.74 (2H, m, −CH_2−_), 0.62 (16H, m, Si-CH_2−_). MS—calculated for C_41_H_76_N_2_O_13_Si_8_, 1029.73; found, 1029.54. Elemental analysis—calculated: C, 47.8%; H, 7.44%; N, 2.72%. Found: C, 48.1%; H, 7.56%; N, 2.29%.

### 4.4. Synthesis of Ln(Q-POSS)_3_ (Ln = Er^3+^, Nd^3+^, Yb^3+^) Hybrid Material

The lanthanide trichloride methanol solution was added dropwise to a Q-POSS toluene solution. The molar ratio of Ln^3+^/Q-POSS was 1:3. The mixture was heated under reflux at 90 °C for 12 h and then cooled to room temperature. The toluene was evaporated in vacuo, and then those complexes were washed with cold methanol for 3 times and dried at 100 °C under vacuum for 12 h. Nd(Q-POSS)_3_ in 58.6% yield (0.12 g), Yb(Q-POSS)_3_ in 53.7% yield (0.11 g), and Er(Q-POSS)_3_ in 60.7% yield (0.13 g). Elemental analysis for Yb(Q-POSS)_3_, calculated: C, 45.3%; H, 6.95%; N, 2.95%. Found: C, 46.4%; H, 6.35%; N, 2.57%; Er(Q-POSS)_3_, calculated: C, 47.8%; H, 7.35%; N, 2.72%. Found: C, 47.0%; H, 7.21%; N, 2.42%; Nd(Q-POSS)_3_, calculated: C, 48.2%; H, 7.42%; N, 2.74%. Found: C, 47.5%; H, 7.36%; N, 2.53%.

### 4.5. Synthesis of the LnQ_3_ Complexes (Ln = Er^3+^, Nd^3+^, Yb^3+^)

The synthesis procedure was according to the procedure described in the literature [27]. The lanthanide trichloride methanol solution was added dropwise to 8-hydroxyquinoline (HQ) methanol solution. The molar ratio of Ln^3+^/Q was 1:3. Upon addition of the chloride to the quinoline solution, the solution became an orange color. The mixture was heated under reflux at 55 °C for 10 h and then cooled to room temperature. After an appropriate amount of water was added, plenty of precipitates appeared and were collected by filtration and washed with cold methanol. Then these complexes were dried at 80 °C under vacuum for 12 h. NdQ_3_ in 31.2% yield (0.18 g), ErQ_3_ in 36.3% yield (0.22g), YbQ_3_ in 53.3% yield (0.32g). Elemental analysis for YbQ_3_, calculated: C, 53.56%; H, 3.00%; N, 6.94%. Found: C, 51.3%; H, 2.93%; N, 6.29%; ErQ_3_, calculated: C, 54.1%; H, 3.03%; N, 7.01%. Found: C, 53.3%; H, 3.03%; N, 6.07%; NdQ_3_, calculated: C, 56.2%; H, 3.15%; N, 7.29%. Found: C, 53.5%; H, 3.34%; N, 7.12%.

### 4.6. Synthesis of Gd(Q-POSS)_3_ Complex

The synthesis procedure was according to the procedure described in the literature [19]. The powder GdCl_3_ was added to Q-POSS toluene solution with a molar ratio of 1:3 (GdCl_3_: Q-POSS), and then the mixture was kept stirring at 90 °C for 12 h in a nitrogen atmosphere. The precipitates were collected by filtration, washed with anhydrous ethanol, and dried at room temperature under vacuum overnight. Gd(Q-POSS)_3_ in 40.3% yield (0.25 g).

## Supplementary Materials

The following are available online, Figure S1: ^1^HNMR spectrum of the Q-POSS ligand. Figure S2: MALDI-TOF mass spectrum of the Q-POSS ligand. Figure S3: UV–Vis absorption spectra of HQ, ErQ_3_, NdQ_3_, and YbQ_3_ in dichloromethane solutions (1 × 10^−5^ M) at room temperature. Figure S4: Excitation spectra of NdQ_3_ and Nd(Q-POSS)_3_ Figure S5: Excitation spectra of YbQ_3_ and Yb(Q-POSS)_3_. Figure S6: Excitation spectra of ErQ_3_ and Er(Q-POSS)_3_. Figure S7: Emission spectrum of the Gd(Q-POSS)_3_ (λ_ex_ = 302 nm) complex at 77 K.
