# Supplementary material for: Synthesis and Near Infrared Luminescence Properties of a Series of Lanthanide Complexes with POSS Modified Ligands
Source: Molecules. 2019 Mar 30;24(7):1253. doi: 10.3390/molecules24071253 (PMC6480010; doi:10.3390/molecules24071253)
Supplement: Supplementary file 1 [file molecules-24-01253-s001.pdf]

molecules

Supporting Information

Synthesis and Near Infrared Luminescence Properties of a Series of Lanthanide Complexes with POSS Modified Ligands

Qingrui Zhang [1,2], Xiuyun Yang [1,*], Ruiping Deng [2,*], Liang Zhou [2,*], Yang Yu [2] and Yunhui Li [1]

1 School of Chemistry and Environmental Engineering, Changchun University of Science and Technology, Changchun 130000, China; qrzhang784783@126.com (Q.Z.); liyh@cust.edu.cn (Y.L.)
2 Changchun Institute of Applied Chemistry, Chinese Academy of Sciences, Changchun 130022, China; yuy@ciac.ac.cn
* Correspondence: yangxiuyun@cust.edu.cn (X.Y.); dengrp@ciac.ac.cn (R.D.); zhoul@ciac.ac.cn (L.Z.); Tel.: +86-431-85262135

The ^{1}HNMR spectrum and MS spectrum of polyhedral oligomeric silsesquioxanes (POSS) modified 8-hydroxyquinoline derivative (Q-POSS) are shown in Figures S1 and S2, respectively. The UV–vis absorption spectra of HQ, ErQ_3, NdQ_3, and YbQ_3 in dichloromethane solutions (1×10^{-5} M) at room temperature are depicted in Figure S3. The excitation spectra of the Ln(Q-POSS)$_3$ and LnQ_3 (Ln = Yb, Nd, and Er) complexes are depicted in Figures S4, S5, S6, respectively. The phosphorescence emission spectrum of the Gd(Q-POSS)$_3$ at 77K is shown in Figure S7.

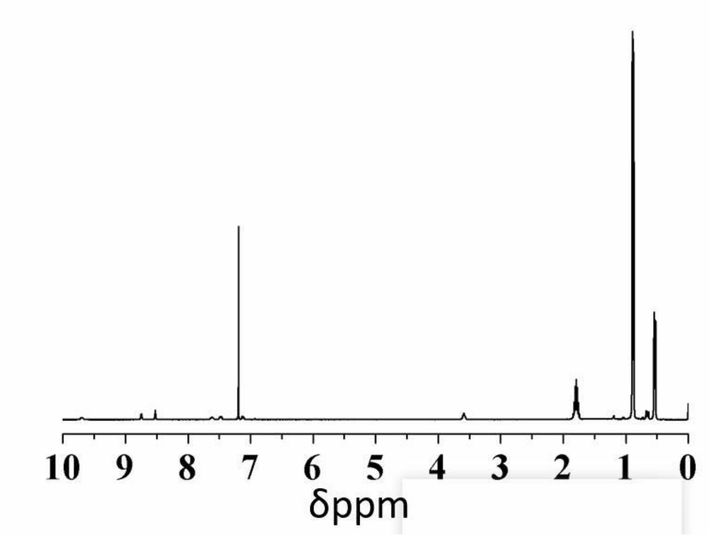

Figure S1. ^{1}HNMR spectrum of the polyhedral oligomeric silsesquioxanes modified 8-hydroxyquinoline derivative (Q-POSS) ligand.

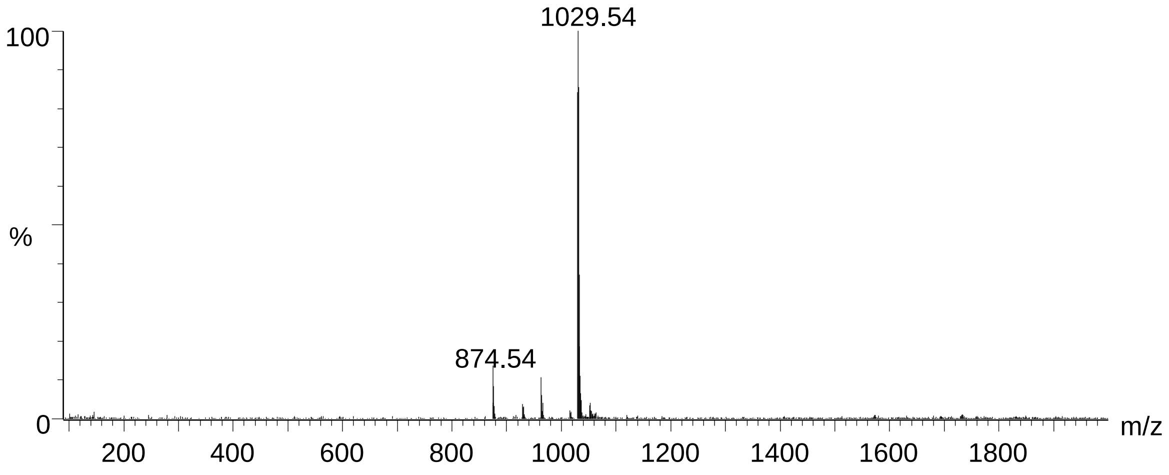

Figure S2. MALDI-TOF mass spectrum of the Q-POSS ligand.

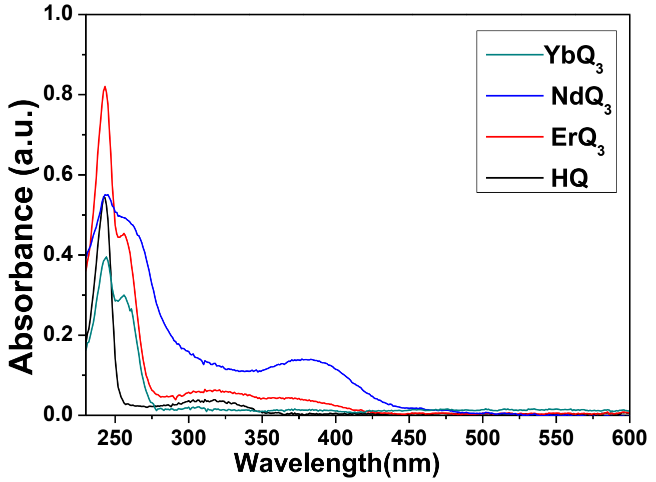

Figure S3. UV−vis absorption spectra of HQ, ErQ_3, NdQ_3, and YbQ_3 in dichloromethane solutions (1×10^{-5} M) at room temperature.

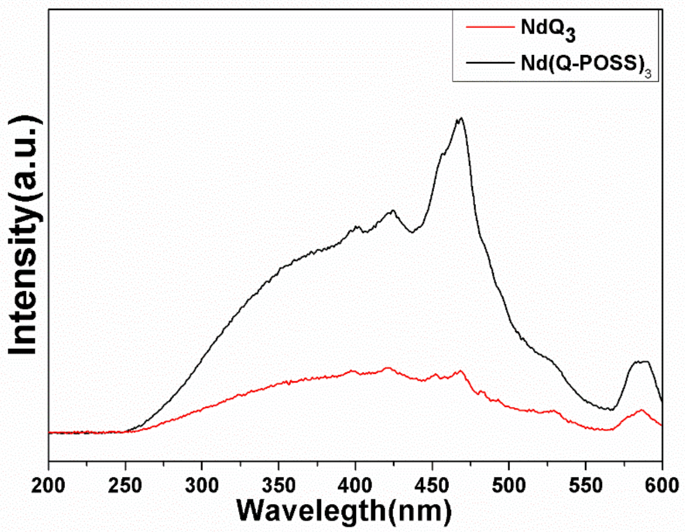

Figure S4. Excitation spectra of NdQ_3 and Nd(Q-POSS)$_3$ (monitored at 1068 nm).

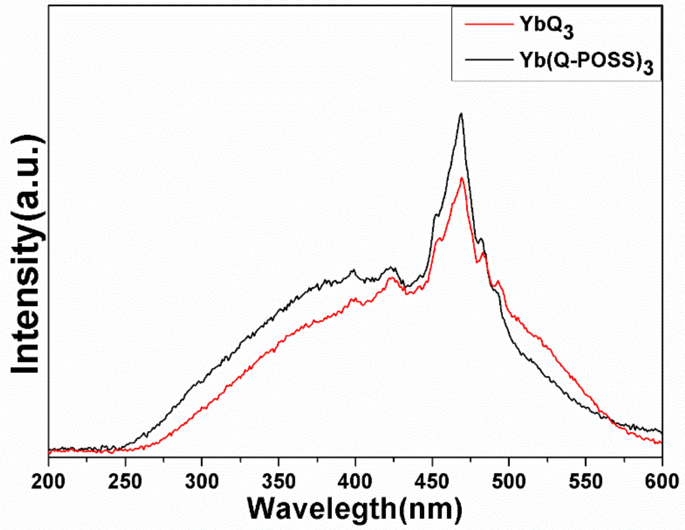

Figure S5. Excitation spectra of YbQ_3 and Yb(Q-POSS)$_3$ (monitored at 980 nm).

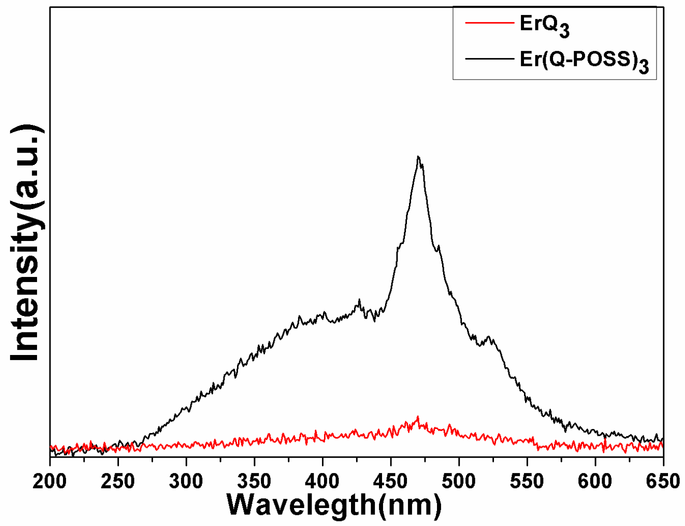

Figure S6. Excitation spectra of ErQ_3 and $Er(Q\text{-}POSS)_3$ (monitored at 1536 nm).

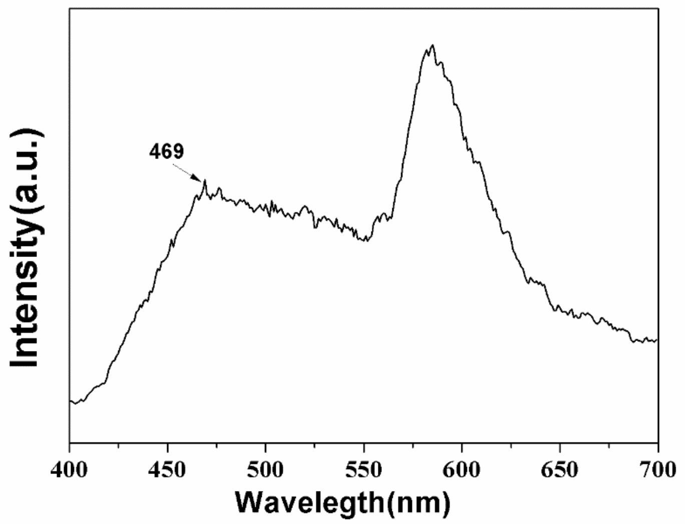

Figure S7. Emission spectrum of the $Gd(Q\text{-}POSS)_3$ complex (λ_{ex} = 302 nm) complex at 77 K.
